# Ensembles of Hydrophobicity Scales as Potent Classifiers for Chimeric Virus-Like Particle Solubility – An Amino Acid Sequence-Based Machine Learning Approach

**DOI:** 10.3389/fbioe.2020.00395

**Published:** 2020-05-05

**Authors:** Philipp Vormittag, Thorsten Klamp, Jürgen Hubbuch

**Affiliations:** ^1^Institute of Engineering in Life Sciences, Section IV: Biomolecular Separation Engineering, Karlsruhe Institute of Technology (KIT), Karlsruhe, Germany; ^2^BioNTech SE, Mainz, Germany

**Keywords:** virus-like particles, solubility, hydrophobicity, hydrophobicity scales, machine learning, feature selection

## Abstract

Virus-like particles (VLPs) are protein-based nanoscale structures that show high potential as immunotherapeutics or cargo delivery vehicles. Chimeric VLPs are decorated with foreign peptides resulting in structures that confer immune responses against the displayed epitope. However, insertion of foreign sequences often results in insoluble proteins, calling for methods capable of assessing a VLP candidate’s solubility *in silico*. The prediction of VLP solubility requires a model that can identify critical hydrophobicity-related parameters, distinguishing between VLP-forming aggregation and aggregation leading to insoluble virus protein clusters. Therefore, we developed and implemented a soft ensemble vote classifier (sEVC) framework based on chimeric hepatitis B core antigen (HBcAg) amino acid sequences and 91 publicly available hydrophobicity scales. Based on each hydrophobicity scale, an individual decision tree was induced as classifier in the sEVC. An embedded feature selection algorithm and stratified sampling proved beneficial for model construction. With a learning experiment, model performance in the space of model training set size and number of included classifiers in the sEVC was explored. Additionally, seven models were created from training data of 24–384 chimeric HBcAg constructs, which were validated by 100-fold Monte Carlo cross-validation. The models predicted external test sets of 184–544 chimeric HBcAg constructs. Best models showed a Matthew’s correlation coefficient of >0.6 on the validation and the external test set. Feature selection was evaluated for classifiers with best and worst performance in the chimeric HBcAg VLP solubility scenario. Analysis of the associated hydrophobicity scales allowed for retrieval of biological information related to the mechanistic backgrounds of VLP solubility, suggesting a special role of arginine for VLP assembly and solubility. In the future, the developed sEVC could further be applied to hydrophobicity-related problems in other domains, such as monoclonal antibodies.

## Introduction

New formats of targeted therapies are emerging, such as virus-like particles (VLPs) (Ong et al., 2017). VLPs are highly immunogenic macromolecular assemblages based on viral proteins, resembling the structure of the virus they were derived from Kushnir et al. (2012). Since they lack viral nucleic acids, the particles are non-infectious (Chackerian, 2007; Kushnir et al., 2012). VLPs are on the market as vaccines against the virus they were derived from, e.g., human papillomavirus-VLPs against human papillomavirus infection to prevent cervical cancer, or hepatitis B surface antigen-VLPs against hepatitis B virus infection (McAleer et al., 1984; Bryan et al., 2016). An approach increasingly investigated is the display of foreign epitopes on a VLP scaffold resulting in chimeric VLPs (cVLPs) (Ong et al., 2017). They benefit from the inherent immunogenicity of a viral structure coupled with the structure of a foreign target antigenic epitope (Pumpens and Grens, 1999). Hepatitis B core antigen (HBcAg) has been widely applied as a VLP platform for chimeric antigen display due to its excellent stability, successful production in a high diversity of expression systems, and induction of strong B- and T-cell responses (Pumpens and Grens, 1999; Jegerlehner et al., 2002; Klamp et al., 2011). The foreign peptide is typically introduced genetically into the VLP at the N-terminus, C-terminus, or preferably in the major immunodominant region (MIR) (Karpenko et al., 2000; Pumpens and Grens, 2001). However, insertion of a foreign epitope often results in insoluble, misassembled or aggregated capsids, lacking the desired immunogenicity (Karpenko et al., 2000; Billaud et al., 2005; Gillam and Zhang, 2018). The process of identifying soluble cVLP constructs is highly empirical and time-consuming (Chackerian, 2007). While few reports studied cVLP solubility based on sequence data, the number of observations included in these studies is limited (Karpenko et al., 2000; Billaud et al., 2005; Janssens et al., 2010). Early development of cVLPs would therefore greatly benefit from a model to predict solubility which is probed using a large data set.

A variety of general approaches to predict protein solubility exists that are based on information from 3-D structures and simulations and/or amino acid sequence information. For a detailed overview of aggregation and solubility prediction tools, we refer to a recent review (Trainor et al., 2017). 3-D structure-based methods include the prediction of soluble expression by molecular dynamics (MD)-simulated unfolding combined with a support vector machine (SVM) architecture (Schaller et al., 2015), dynamic exposure of hydrophobic patches in MD simulations (Chennamsetty et al., 2009; Jamroz et al., 2014), and projection of sequence-based methods onto 3-D structures (Sormanni et al., 2015; Zambrano et al., 2015). Although high-throughput 3-D structure generation of VLP building blocks has been described previously (Klijn et al., 2019), the computational cost of creating 3-D structures is still high, limiting the applicability of this approach in candidate selection for several hundred molecules. Amino acid sequence-based methods can be distinguished into amino acid composition-based algorithms such as machine learning approaches using SVM or random forest classifiers (Magnan et al., 2009; Agostini et al., 2012; Samak et al., 2012; Xiaohui et al., 2014; Yang et al., 2016) and sliding-window-based algorithms, such as AGGRESCAN, Zyggregator, and CamSol (Conchillo-Sole et al., 2007; Tartaglia et al., 2008; Sormanni et al., 2015). Interestingly, sequence-based methods have been reported to be superior to solvent-accessible surface-based methods in the prediction of monoclonal antibody aggregation (Hebditch et al., 2019).

The above-mentioned methods have in common that their goal is to identify proteins or patterns in proteins that are prone to aggregation and therefore have a higher chance to be insoluble upon expression. In the following section, we will discuss why hydrophobic interactions play a special role for VLP solubility and why the application of current models is difficult for the cVLP solubility problem.

HBcAg has been extensively studied in a C-terminally truncated form with amino acids 150–183 removed, termed Cp_1__–__149_. It assembles to VLPs while being easier to handle in experiments and processes than the full-length HBcAg. This is attributed to the removal of the strongly positively charged C-terminal amino acids that bind nucleic acids (Gallina et al., 1989; Zlotnick et al., 1996; Wizemann and von Brunn, 1999; Alexander et al., 2013). The smallest HBcAg species observed in physiological solutions are dimers, stabilized by an intermonomer disulfide bridge and a hydrophobic core (Wynne et al., 1999). The dimeric Cp_1__–__149_ aggregates aggressively and readily forms capsids at low concentrations, neutral pH, and low salt (Ceres and Zlotnick, 2002). Capsid formation is an entropy-driven process relying on hydrophobic interaction and is therefore similar to protein aggregation (Ceres and Zlotnick, 2002; Gorbenko and Trusova, 2011). Since capsids can exist in much higher concentrations in physiological buffers than dimers, the solubility limitation introduced by insertion of a foreign epitope is most probably related to an interference with the assembly reaction (Billaud et al., 2005; Chackerian, 2007). Investigation of chimeric HBcAg VLP assembly by diafiltration showed a dependence of the assembly reaction on the inserted epitope sequence (Rüdt et al., 2019). When the assembly reaction is hampered by the insertion of a foreign epitope, the strong entropic drive for protein-protein interaction probably leads to insoluble aggregates as opposed to soluble capsids. Ordered aggregation of dimers to capsids can therefore be assumed as a prerequisite for high-level soluble expression, whereby hydrophobicity plays a major role. Therefore, an appropriate measure of hydrophobicity is paramount to describing the cVLP solubility problem.

The hydrophobic effect is described by the free energy change of water surrounding a solute. For amino acids in specific, it has been investigated based on organic solvent-water partition coefficients, for example (Nozaki and Tanford, 1971). This partition coefficient might be suitable for the description of solute distribution in such systems. Protein folding, however, is a much more complicated matter influenced by more and different properties of the amino acids than their tendency to accumulate in a certain phase and is still not fully understood (Garde and Patel, 2011; Harris and Pettitt, 2016). To overcome the limitation of this definition of hydrophobicity for biological systems, so-called hydrophobicity scales have been developed. These are, for example, based on the analysis of the distribution of amino acids in the core or surface of the protein (Naderi-Manesh et al., 2001), thermodynamic calculations elevating different aspects of solvation (Chothia, 1976; von Heijne and Blomberg, 1979), or peptide retention times in reversed-phase chromatography (Wilce et al., 1995). These scales have in common that they try to describe the hydrophobic effect in the interplay with other factors related to geometries and electrostatic contributions. It is therefore important to note that throughout this manuscript, hydrophobicity is referred to as a value describing the tendency of proteins, amino acids, or functional groups to influence a biological process toward an outcome that is thought to be connected with hydrophobicity, such as aggregation, rather than its strict thermodynamic definition for smaller solutes (Harris and Pettitt, 2016). Hydrophobicity scales assign each proteinogenic amino acid a particular hydrophobicity value. These hydrophobicity values can be used to calculate overall protein hydrophobicity or regions within the molecule. Simm et al. (2016) identified 98 protein hydrophobicity scales in the literature. These scales have been derived using experimental and theoretical techniques based on a great variety of training data, ranging from small to large sets of proteins, peptides, single amino acids, or 3-D structures. Application of a hydrophobicity scale to a new problem requires that an appropriate scale is chosen. This can be based on comparison of the investigated experimental conditions to the framework in which the hydrophobicity scales were derived or the choice of frequently applied hydrophobicity scales. None of these two approaches is recommended as they both introduce bias into the model. Feature selection algorithms can help overcoming this bias and selecting the appropriate scales using a set of training data. In a study on aggregation-prone regions of 354 peptides, feature selection has been successfully employed to derive critical features for peptide or protein aggregation (Fang et al., 2013). The most important 16 critical features were incorporated in SVM and random forest architectures. In another study, an SVM architecture using 40 features was applied (Tian et al., 2009). These methods project the problem onto a space of a dimension of the number of feature variables. This could also be applied to hydrophobicity values calculated by several hydrophobicity scales. Another approach is to regard each hydrophobicity scale individually to be included in a classifier in a one-dimensional input data space. Reflecting upon hydrophobicity scales, this is reasonable since each of the scales were derived to be individual measures of hydrophobicity. Considering them individually, the physicochemical meaning behind the scales remains largely unchanged. The strength of this method comes with the combination of several classifiers. This results in potent ensembles that incorporate the classifiers’ strengths, while ideally overshadowing their weaknesses in classification (Matteo and Giorgio, 2012). In an article on hydrophobicity scale optimization by a genetic algorithm, the authors pointed out, that statistical methods may have strong prediction performance, but may not be applicable to new or even to similar problems and small data sets (Zviling et al., 2005). Therefore, preserving physicochemical information in hydrophobicity scales was one important goal in this research.

In summary, ensemble methods based on hydrophobicity scales promise to be a potent tool to describe classification or regression problems related to hydrophobicity. The cVLP solubility problem calls for a method that ascertains critical features of the molecules in an aggregating environment, capable of distinguishing between structures that probably aggregate to soluble VLPs and those that aggregate to insoluble structures. The objective of this study was to create an interpretable protein solubility model framework and to uncover information about the VLP solubility problem that will aid in engineering soluble cVLP candidates. Therefore, a soft ensemble vote classifier (sEVC) was developed and implemented, which consists of individual decision trees, each based on a hydrophobicity scale including an embedded feature selection algorithm. Physicochemical information contained in the hydrophobicity scales was largely conserved by (I) using each scale as an individual classifier within an ensemble and (II) by implementing a simple one-level decision tree as classifier. Feature selection was implemented to boost model performance and identify the most relevant hydrophobicity scales for chimeric HBcAg VLP solubility. The applicability of the model was evaluated with 568 chimeric C-terminally truncated HBcAg VLP constructs using 91 hydrophobicity scales.

## Materials and Methods

### VLP Solubility Data

Chimeric HBcAg VLP constructs were based on His-tagged C-terminally truncated HBcAg (Schumacher et al., 2018). The molecules were created using 82 different peptide inserts and eight different insertion strategies, a total of 691 chimeric VLP constructs, which were experimentally tested for solubility. The peptides are inserted into the HBcAg molecule in the MIR. An insertion strategy defines where exactly in the MIR the peptide is inserted and which amino acids are deleted. Inserts that have not been tested with all eight insertion/deletion strategies were excluded from this study. The final data set comprised 568 chimeric HBcAg VLPs with all possible combinations of strategies A-H and inserts 1–71. Solubility was evaluated by SDS-PAGE after lysis of the expression host *E. coli*. Solubility was treated as a binary class system with class labels “soluble” or “positive” or “1” and “insoluble” or “negative” or “0”. Throughout this paper, an “observation” is referred to one of the 568 chimeric HBcAg VLP constructs. Class “soluble” was attributed to 283 of 568 observations, while 285 of 568 were class “insoluble.” With 49.8%/50.2% class division, the data set can be considered as a balanced classification problem.

### Data Set Division

Model training, model evaluation, and data processing were performed with MATLAB R2018a (The Mathworks, Natick, US-MA). Models were always generated by and calculated on randomly selected validation subsets (or with stratified random sampling). In this article, randomization is achieved by using the *randn* command of MATLAB, which generates pseudorandom values. Seven data subsets containing *n*_train_ observations were created prior to model construction, where *n*_train_ = {24,24,24,48,96,192,384}. These data sets were constructed once and the remainder of available data *n*_test_ was used as an external test set. Observations were drawn from the data set by stratified sampling aiming at a balanced representation of strategies and inserts, i.e., the respective strata. Stratified sampling was achieved by limiting the occurrence of strategies and inserts in the data set. The maximum allowed number of inserts in the sampled data set was $n_{\text{insert},\max}=r\,o\,u\,n\,d\,u\,p\,\left(\frac{n_{\text{train}}}{n_{\text{inserts}}}\right)$, where *n*_inserts_ = 71. The maximum allowed number of strategies was accordingly $\frac{n_{\text{train}}}{n_{\text{strategies}}}$, where *n*_strategies_ = 8. When the maximum number of a certain insert or strategy in the training set was reached, all identical inserts or strategies, respectively, were made unavailable to random selection in order to sample the strata evenly.

### Hydrophobicity Scales

Ninety eight hydrophobicity scales were retrieved from a recent article on peptide classification (Simm et al., 2016), originally derived from AAindex (Kawashima et al., 2007), the SPLIT 4.0 server (Juretiæ et al., 1993), and ProtScale (Gasteiger et al., 2005). Each scale was centered and scaled to unit variance. Reversed scales were excluded if there was a complementary, non-reversed scale available, resulting in 91 scales (see Supplementary Table S1).

### Hydrophobicity Scale-Based Soft Decision Tree Ensemble Vote Classifier

**FIGURE 1:**
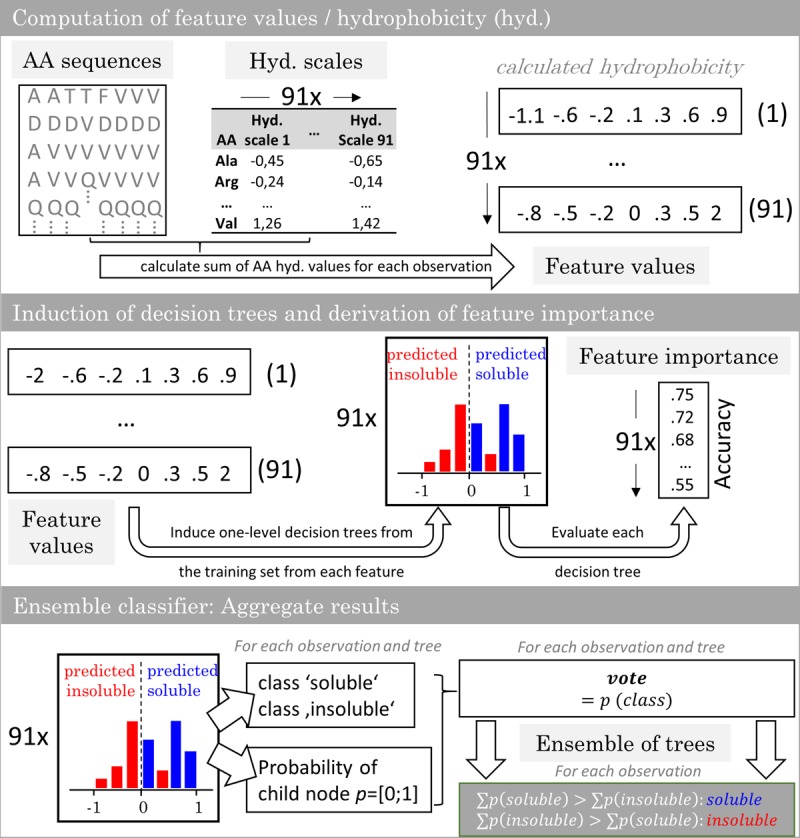
Workflow of the ensemble vote classifier. The ensemble vote classifier is constructed by computation of feature values from virus-like particle sequence data and 91 hydrophobicity scales. With the training set features, one-level decision trees are induced. The individual decision trees’ accuracy in predicting the training set is defined as the feature importance. In the ensemble model, each decision tree contributes a solubility decision with associated probability. The results are aggregated and the most probable class is chosen by the ensemble.

The model generation comprised a feature selection, a soft ensemble vote classifier (sEVC) informed by classifiers based on hydrophobicity scales, and a Monte Carlo cross-validation (MC-CV) procedure. Figure 1 illustrates the construction of the sEVC. Feature values were computed from amino acid sequences and hydrophobicity scales. A hydrophobicity scale assigns each amino acid a hydrophobicity value. The sum over the amino acids results in the feature value 0.73 amino acids in N- and 71 amino acids in C-terminal direction were omitted in the calculation, as they were identical for all constructs. Each classifier in the sEVC was constructed from feature values calculated for each observation in the training set using one hydrophobicity scale. The individual classifiers based on this feature value were decision trees with one split (also called decision stumps) which were trained based on Gini’s diversity index (Gini, 1912; Windeatt and Ardeshir, 2004). This one-level tree design ensures that a simple hydrophobicity threshold decides about the predicted class. Decision trees were constructed using the *fitctree* function of MATLAB’s *statistics and machine learning toolbox*. The resulting *n*_trees_ decision trees assigned each observation a class decision and a class probability. For a hard ensemble vote classifier, the probabilities are equal to 1. In the here applied sEVC, the class probability is the probability estimate derived from the associated child node in the decision tree. The class decision and the associated class probability becomes the decision tree’s *vote*. The *votes* of all decision trees for a particular observation are summed up in the sEVC. The class that has a higher sum of probability values is the elected class.

**FIGURE 2:**
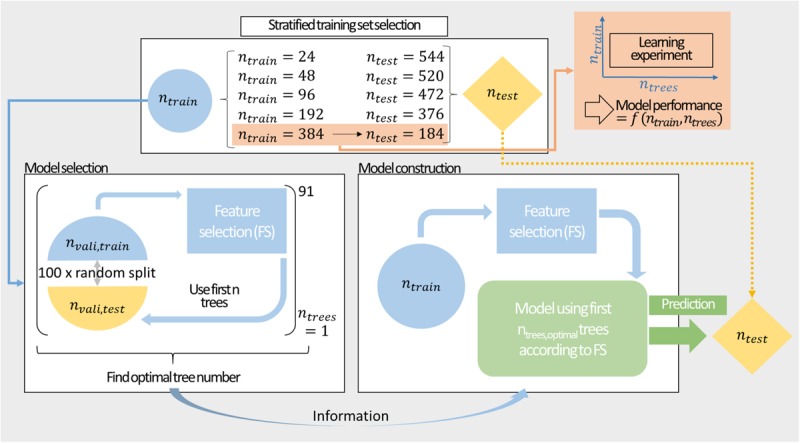
Modeling workflow comprising stratified sampling, a learning experiment, model selection, and construction. Stratified sampling results in training sets of *n*_train_ = {24,48,96,192,384} data points. These training sets are split in 100-fold Monte Carlo cross-validation to inform about the optimal number of classifiers. The training set is then used to construct a model with preceding feature selection to predict the external test set. The largest training set is additionally utilized for a learning experiment exploring the performance of the model in the space of training set size and number of included decision trees.

Figure 2 shows the procedure for model construction from stratified training set selection, over model selection by MC-CV through to model construction and prediction. Model performance was evaluated by 100-fold MC-CV. During validation, 50% of the data was used for training and the remaining data was predicted. MC-CV samples *n*_*vali,train*_ randomly without replacement. Compared to k-fold cross-validation, the number of cross-validation groups in MC-CV is not governed by the choice of their sizes, and observations can be sampled in different cross-validation sets. The information on the model performance can then be used to inform about optimal classifier numbers for construction of the model. For the final model, the entire training data set is used for model training and feature selection. The embedded feature selection sorts the features with decreasing feature importance. In 91 models, the best 1–91 classifiers are included. The resulting classifiers are used to predict the external test set.

### Model Performance Evaluation

The performance of the sEVC was evaluated based on Matthew’s correlation coefficient

(1) $$\begin{array}{c} M\,C\,C=\frac{T\,P\times T\,N-F\,P\times F\,N}{\sqrt{\left(T\,P+F\,P\right)\,\left(T\,P+F\,N\right)\,\left(T\,N+F\,P\right)\,\left(T\,N+F\,N\right)}} \end{array}$$

where *TP*, *TN*, *FP*, and *FN* stand for true positive, true negative, false positive, and false negative classification of the model subsets, respectively (train, validation, and test contingency matrix). The MCC is considered to be the least biased singular metric to describe the performance of binary classifiers, especially for cases of class imbalance (Powers, 2011; Chicco and Jurman, 2020). Another metric that was used is the accuracy *A* as defined in Equation (2).

(2) $$\begin{array}{c} A=\frac{T\,P+T\,N}{T\,P+T\,N+F\,P+F\,N} \end{array}$$

### Feature Selection

The model generation was preceded by an embedded feature selection. The decision trees were evaluated individually to assess feature importance (Figure 1). The feature importance was defined as the accuracy of the individual decision trees for the prediction of the training set. While the MCC is a less biased metric (Powers, 2011), it is not defined for cases where terms in the denominator are zero, which was the case for the smallest training sets. For comparability, accuracy was subsequently used as the feature importance metric throughout this study. Feature importance was computed for every model and for each validation run. The features (and thus the respective decision trees) were then sorted in descending order according to their importance, so that most important features were chosen first during model generation.

### Learning Experiment

To explore the model design space with further scrutiny, the model’s performance was characterized in a learning experiment. To investigate the effect of training set size, the number of training observations was varied in steps of 5% of the *n*_train_ = 384 data set from 5 to 95%, resulting in 19 different training set sizes (see also Figure 2). The external test set was composed of the remaining 184 observations. The training sets were drawn randomly without stratified sampling from the stratified *n*_train_ = 384 data set. The remainder of the 384 observations was not used or evaluated. The number of included decision trees (1–91) was screened in addition to the training set size. Thus, a matrix of 19 × 91 individual model settings was created. Each model setting was repeated ten times resulting in 19 × 10 × 91 models. Each training set was sampled individually for all 19 × 10 × 91 models, resulting in 17,290 training sets. Feature selection was performed 17,290 times, i.e., individually for each of the models. Model performance was evaluated based on the external test set and the training set. The median and median absolute deviation (MAD) of the ten model repetitions were computed. They were the basis for the discussion of model performance at respective training set sizes and included number of decision trees.

### Systematic Misclassification

To evaluate systematic misclassification, the frequency of true and false predictions were evaluated for each insertion strategy. The relative frequency of strategies found within the classification groups *TP*, *TN*, *FP*, and *FN* was calculated by summing up their occurrence in the respective groups in the 17,290 models of the learning experiment and normalizing it by the overall occurrence of the strategies in all classification groups and all models.

### Model Generation

The sEVC workflow comprises stratified training set selection, model validation by MC-CV and prediction of an external test set (Figure 2). The number of included decision trees was a hyperparameter that was screened for the model generation on the *n*_train_ = {24,24,24,48,96,192,384} data sets. The optimal number of included decision trees in the MC-CV validation procedure should inform about the best model for the prediction of the external test set. This relationship was investigated for all seven training sets.

## Results and Discussion

### Data Set Construction

**FIGURE 3:**
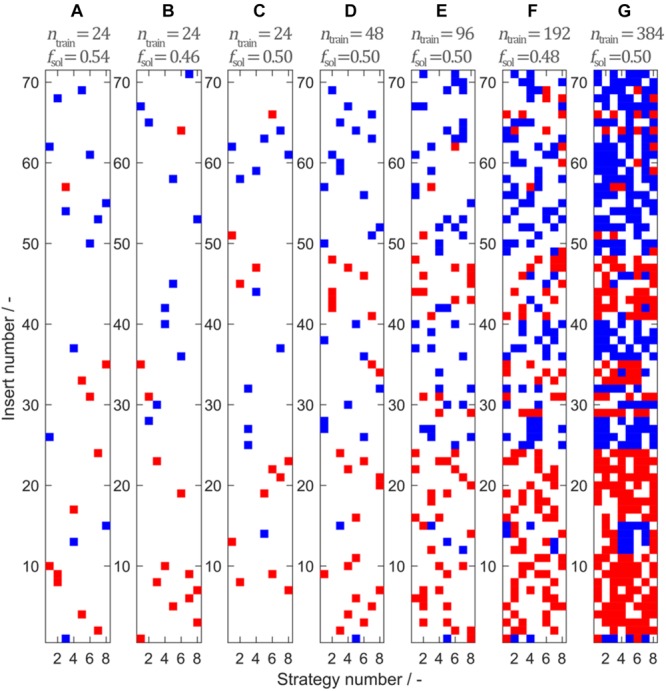
Model training sets 1–7 **(A**–**G**) created by stratified sampling of *n*_train_ = {24,24,24,48,96,192,384} data points. Stratified sampling was informed by the construction of the entire data set, where eight insertion strategies were used for 71 different inserts, amounting to a total of 568 observations. While for the sampling procedure the solubility data of the observations were unknown, their solubility class is illustrated for interpretation purposes. Blue represents soluble and red represents insoluble observations. The fraction of soluble observations is indicated above the plots by *f*_sol_.

The data set consists of observations that can be assigned to 71 unique peptide inserts and 8 unique insertion strategies. Stratified sampling was used to build a representative training set from the full data set. Figure 3 shows seven training sets comprising *n*_train_ = {24,24,24,48,96,192,384} observations sampled by 2-D stratified sampling in a grid of inserts over insertion strategies. Soluble constructs are marked in blue and insoluble constructs are marked in red. The fraction of soluble constructs in the training set *f*_sol_ is between 0.46 and 0.54, resulting in a maximum deviation of 0.04 from the expected value *f*_sol__,total_ = 0.498. In the seven models, deviation of *f*_sol_ from the theoretically expected value of *f*_sol__,total_ = 0.498 derives from random sampling but is limited due to stratified sampling. From Figure 3G, it can be seen that the choice of the insert is strongly influencing solubility, while the insertion strategy only has an effect on solubility for a small number of constructs. This pattern is confirmed when considering the entire solubility matrix (Separate Data Table in the Supplementary Material), underpinning the usefulness of stratified sampling especially for smaller data sets. With 24 training examples, only a third of the 71 inserts are represented by the training set. To investigate the influence of this potential lack of information during model training, three different training sets with 24 samples have been created.

### Influence of Training Set Size and Number of Decision Trees in the Ensemble Vote Classifier

**FIGURE 4:**
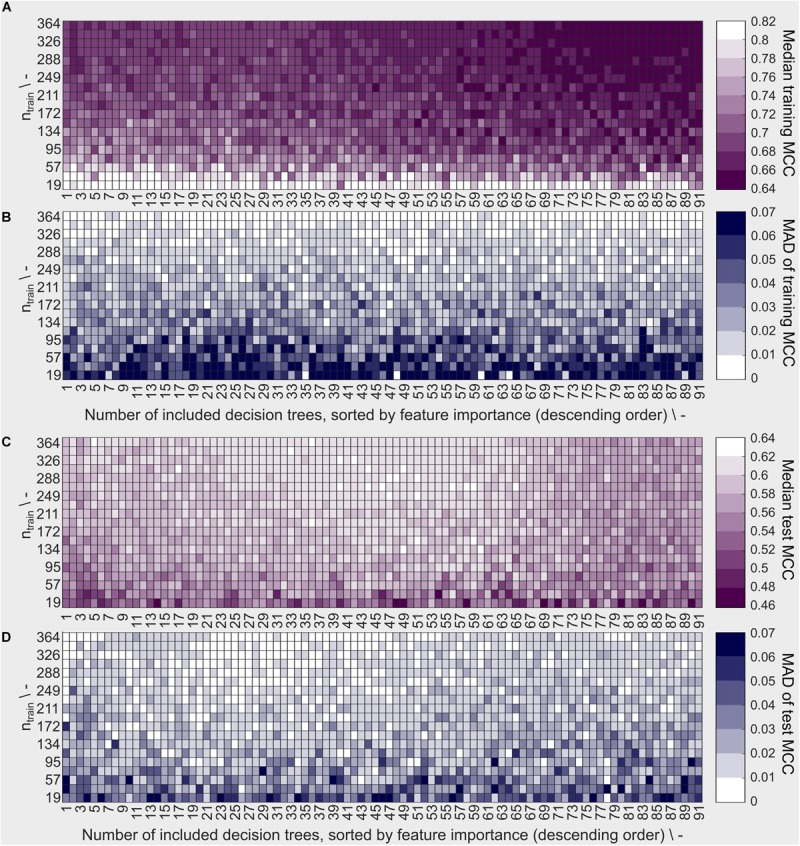
Model performance based on training and external test data described by **(A)** the median Matthew’s correlation coefficient (MCC) and **(B)** the median absolute deviation (MAD) of the MCC for training data and **(C)** median MCC and **(D)** MAD of MCC for external test data. Each rectangle represents a decaplicate of a model with the number of training examples shown on the y-axis and the number of included decision trees in the ensemble classifier on the x-axis. The training observations were randomly sampled from the stratified dataset with *n*_train_ = 384. The decision trees are sorted in descending order by feature importance. This means, that at point *n* on the x-axis, the results of the models including the *n* best decision trees are shown. White/bright color denotes high median MCC values and low MAD of the MCC, dark (violet or blue) color denotes low median MCC values and high MAD of the MCC, relative to all MCC data in the learning experiment. A well-predicting and reproducible model has high MCC and low MAD, respectively (both bright).

The sEVC’s performance characteristics were evaluated in a learning experiment exploring the space of training set size and number of included classifiers, i.e., decision trees. In this experiment, 190 × 91 models were created using the best 1–91 decision trees as determined individually for each model by the feature selection algorithm. The highest median training MCC can be observed at low training set sizes of ≤57 (Figure 4A). Note that a brighter color corresponds to better model performance (higher MCC) and lower model variability (lower MAD of MCC). Most models with an MCC > 0.80 are found at the smallest training set size of 19. This concurs with the area where the MAD is greatest with ≥ 0.06 (Figure 4B). Larger MAD values indicate greater variation between the model repetitions. This suggests a high dependency of model performance on the individual random sampling of the training set. Increasing training set size results in lower training MCC and MAD of MCC. MAD is smaller since more information is available during model training. Additionally, large training sets have a higher probability to contain a significant fraction of identical training observations in the ten different random samplings.

Decision trees with lowest feature importance are included in the models with the largest number of included decision trees due to feature selection. Model performance aggravation due to inclusion of these decision trees was the case for larger training sets, where median training MCC decreases with the number of included decision trees.

The external test set observations are identical for all models, while the training set and therefore the resulting model is individually different. Median test set MCC is <0.48 for low training set sizes *n*_train_ ≤ 38 (Figure 4C). Most models of this size produce a test set median MCC of ≤0.54, compared to training MCC of ≥0.8 for most models, suggesting an overfitted model for small training data sets. The largest MCC of the external test set predictions are found at training set sizes ≥ 249 and at > 23 and < 65 number of included decision trees, which is also overlapping largely with the region of lowest MCC MAD with many models showing a test set MAD ≤ 0.01 (Figure 4D). Therefore, in this area, the best models are found having high MCC (most ≥ 0.6) on the test set and low MAD of MCC. The difference between training and external test set MCC in this area is ≤ 0.1. Thus, the training set is a good indicator of model performance in said area.

It has to be noted that the model performance was evaluated on randomly chosen subsets of the stratified *n*_train_ = 384 data set, constraining the benefits from stratified sampling. Stratified sampling can be expected to decrease overfitting and variation seen for low training set sizes, as the probability of drawing a non-representative sample set is drastically reduced, as discussed both above and below.

### Selection of Models Based on Stratified Training Sets

**FIGURE 5:**
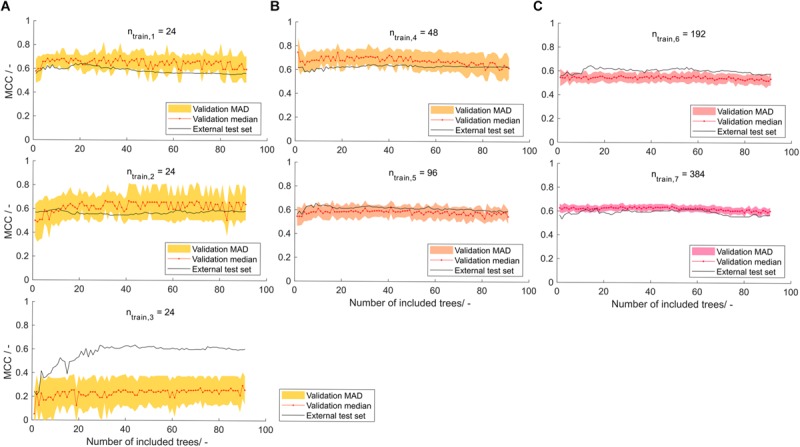
Validation and external test set Matthew’s correlation coefficient (MCC) on stratified training data with training set size of **(A)** 24, **(B)** 48 and 96, and **(C)** 192 and 384 observations, depending on the number of included decision trees. Validation median and median absolute deviation (MAD) are calculated from 100-fold Monte Carlo cross-validation. The median is shown as red dots connected by a line to guide the eye. The shaded area represents the MAD around the median and are colored from yellow over orange to pink with increasing training set size. The black line represents the performance of the model on the external test set of size *n*_test_ = 568−*n*_train_.

In the seven models created by stratified samplings, the shaded area, representing the model’s MAD of validation MCC, is decreasing with increasing training set size (Figure 5). This was expected due to the increasing amount of information available during model training. The sets with *n*_train_ = 24 were constructed three times to investigate the robustness of small training set sizes. Of these, the first has a smaller MAD area than the other two, which can possibly be attributed to a “lucky” stratified sampling. With only 24 samples, the MC-CV comprises 12 validation training and 12 validation test observations, resulting in potentially larger artifacts of random selection. Also, stratified sampling based on the insert does not have a strong effect, since only a maximum of 24 different inserts of the total 71 inserts are chosen, leaving 47 inserts unrepresented. Validation MCCs are comparably stable over the number of included decision trees. Some of the models show a slight MCC increase over the first number of included decision trees (models 1–3 and 5) and some show a gradual but shallow decline at larger numbers of included decision trees (>50; models 4, 6, and 7). This underlines the effect seen in the learning graph while being less pronounced, which probably can be attributed to the more balanced training data set. Most of the models result in validation MCCs of around 0.6, whereas model 3 of training set size *n*_train_ = 24 has a significantly lower validation MCC of around 0.2. When considering the MCC of the external test sets, each of the models shows adequate performance when a minimum number of decision trees was included. At around 30 decision trees, the models have an external test set MCC that is either above or close to their overall median MCC. The test set MCCs at 30 decision trees are > 0.6 for all models, except model 2 with an MCC of 0.56. The mean MCC of all seven models’ external test data over decision tree numbers was computed to inform about the average model performance dependent on the number of included decision trees (data not shown). It was optimal at 29 and 30 decision trees, both with a mean MCC of 0.61.

At larger training set sizes, the trend in validation data is more translatable to the trend in external test data, while at low training set sizes of 24 observations, the model should only partially rely on validation data and may include a minimum number of decision trees to avoid overfitting. Another reason for this is feature selection, which is performed on a potentially unrepresentative data set. It can therefore result in prioritizing decision trees that fit unrepresentative data but not the entire data set well. Comparison of the model’s performance to other published models can be difficult since many report their results as accuracies – typically in the range of 0.62–0.83 (Idicula-Thomas et al., 2006; Smialowski et al., 2006; Magnan et al., 2009; Hebditch et al., 2017). In the ideal balanced case, the MCC of these models would be 0.24–0.66 (for the explanation on the relation of MCC and accuracy see Supplementary Material S2). However, many of those models are not based on a balanced data set, which would then lead to a lower MCC. The model presented in this paper shows MCC values close to the best MCC estimates of previously published models. In a review on HBcAg cVLPs, insert charge was described to be the most important parameter for solubility of cVLP candidates (Whitacre et al., 2009). Construction of a decision tree on a scale that rates aspartic acid and glutamic acid with -1, arginine and lysine with +1, and all other amino acids with 0, resulted in an MCC of 0.38 on the external test set using the *n*_train_ = 384 training and corresponding test set (data not shown). The correlation of insert charge to cVLP solubility, as described in the above-mentioned review, was therefore observed with the data set investigated in this article. It was not as strong as the predictions of the sEVC based on hydrophobicity scales.

Some trends with regard to the number of included decision trees have been uncovered and discussed. However, it has to be noted that the effects are quite small over a wide range of included decision trees, especially with stratified training sets. On the one hand, this indicates that the model performs well over a large space of a chosen number of classifiers and training set sizes. On the other hand, it highlights the potential of the ensemble classifier to include more orthogonal scales that could describe more aspects in the data and therefore result in even better models. In a very simple way, the orthogonality of the scales can be analyzed by principal component analysis (PCA). PCA on the 91 normalized hydrophobicity scales revealed that the first principal component already explains 68.8% of the variance (Supplementary Figure S1). This may be expected, when considering how the scales were derived. Many of the hydrophobicity scales originate in some way from other scales being only slightly modified. It would therefore be highly interesting to investigate the sEVC framework constructed with a set of scales that complement each other to explain more of the variance found in the data and result in even better models.

### The Potential of Feature Selection to Retrieve Biological Information

**FIGURE 6:**
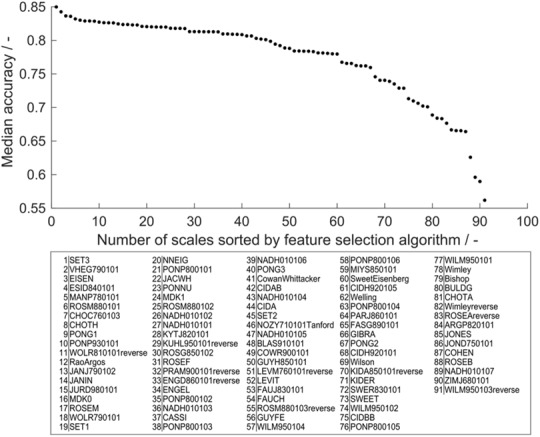
Median feature importance measured by the median training set accuracy of the individual scales in all 190 × 91 models in the learning experiment. Median accuracy of scales was sorted in descending order, so that scale 1 has highest accuracy and therefore highest feature importance, while scale 91 has lowest feature importance. The IDs of the scales (i.e., feature names) are noted below in respective order.

Feature selection is an important tool to boost model performance. It can also serve to retrieve biological information with respect to the modeled problem. Accuracy was chosen as metric for feature importance to avoid cases where the MCC is not defined. Decision trees that individually classify more observations of the training set correctly therefore have higher feature importance. In the learning experiment, 19 different training set sizes (*n*_train_ = 19, 38, …, 384) were evaluated 910 times, giving a statistically strong insight into feature importance in the range of tested training set sizes. Median feature importance ranges from 0.54 to 0.85 (Figure 6) and shows an MAD of 0.02–0.04, while MAD increases toward lower accuracies (data of accuracies and MAD in Supplementary Table S2). This median feature importance value is valid for the entire training data set of 384 observations. It describes, in the framework of the presented chimeric HBcAg VLP solubility problem, which decision tree, and therefore hydrophobicity scale, is most suitable for the distinction of soluble and insoluble constructs independent of the training set size.

Feature importance can be used to obtain a biological interpretation of the model based on the characteristics of the hydrophobicity scales with highest feature importance (best) and lowest feature importance (worst). Feature importance describes their quality to predict within the cVLP solubility problem. With the first three scales, feature importance declines more than with the following 57 scales. The last four scales decrease markedly in feature importance. The best scale SET3 originates from a study on the prediction of transmembrane helical regions (Zviling et al., 2005). In this study, a genetic algorithm optimization approach amplified hydrophobicity and hydrophilicity of hydrophobic and hydrophilic amino acids, respectively, compared to the input scales. Insoluble expression results from protein-protein interaction leading to the formation of aggregates, potentially leading to inclusion bodies (Carrio and Villaverde, 2005). Transmembrane regions in proteins are naturally hydrophobic (Silverman, 2003) and in the absence of membranes therefore prone to protein-protein aggregation. The good performance of this scale suggests that findings on hydropathy based on the propensity to form transmembrane helices is comparable to the solubility investigated in this study. The scale VHEG790101 has second-highest feature importance and was derived using surface accessibility data from Chothia’s study of 1976 (Chothia, 1976; von Heijne and Blomberg, 1979). Free transfer energies of residues from polar to non-polar solvent were calculated adding protonation energies for charged residues. This highlights the benefit of amplifying hydrophilicity of charged residues for application in the VLP solubility scenario. In a machine learning study on protein aggregation, VHEG790101 was also rated as an important feature to predict aggregation propensity (Fang et al., 2013). The EISEN scale has similar but slightly lower feature importance, which can be explained by the fact that its hydrophobicity values are simply the average of five scales’ normalized hydrophobicity values among which is the scale of Chothia and Von Heijne (Eisenberg et al., 1982).

WILM950103reverse, the scale that has lowest feature importance, is based on C4 reversed phase chromatography retention times of peptides (Wilce et al., 1995). Retention on chromatography columns is often based on small fractions of the molecular surface and cannot directly be translated to properties related to the entire molecule (Hebditch et al., 2019). ZIMJ680101 was created by statistical analysis based on only 40 proteins. The space of applicability of this scale is probably limited and it therefore performs badly in the VLP solubility scenario. In the above-mentioned study by Fang and colleagues, ZIMJ680101 also was rated as an important feature (Fang et al., 2013), highlighting that a direct comparison between the presented model and Fang’s model cannot easily be drawn. The third-worst scale is one of seven hydrophobicity scales derived from a study on solvent-accessibility of amino acids (Naderi-Manesh et al., 2001). Scales NADH010101-7 are created by information theory and represent the self-information derived from different thresholds of solvent-accessibility. These aim to describe the amino acids’ surface accessibility within a protein. From scales 1 to 7, the threshold for surface accessibility was increased from 5 to 50% of its maximum accessibility. Scales 1 and 2 with 5 and 9% threshold ranked at 27 and 26 in feature importance analysis. Increased thresholds of 16, 20, 25, and 36% resulted in feature importance ranks of 36, 43, 47, and 39. Scale 7 with 50% accessibility threshold is significantly worse with position 89 of 91 scales in feature selection. This comparison suggests that there is a dependence of the threshold set in the study by Naderi-Manesh and colleagues on the performance of these scales in the solubility model. The scales with low threshold (5 and 9%) only count residues as inaccessible if they are almost completely buried, thus boosting the hydrophobicity of very hydrophobic amino acids relative to the other amino acids. On the contrary, with the cut-off of 50% accessibility, amino acids that have a significant share of solvent-accessible surface but still below 50% will be regarded as hydrophobic. The lower threshold is probably more applicable to the cVLP solubility problem, since the aim of the epitope design is to expose rather than bury it. This in turn means that the insertion of typically strongly buried hydrophobic residues can corrupt protein folding by their orientation to a protein core, potentially leading to misfolded proteins that aggregate to insoluble clusters. The feature importance ranking of these scales might indicate that, for solubility, strongly hydrophobic residues have a significant influence on solubility, while residues that are somewhat hydrophobic, but still have some solvent-accessible area are not as critical. This argument also supports that chromatography-based scales may not be the best choice to describe macro-properties such as solubility, as discussed above.

**FIGURE 7:**
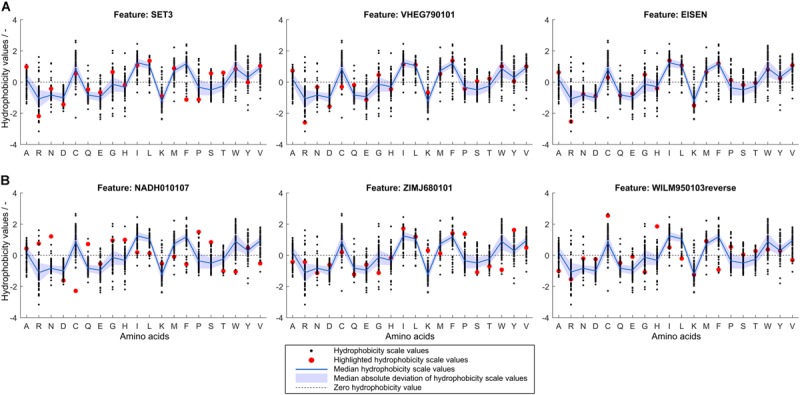
Normalized hydrophobicity scale values for the 20 proteinogenic amino acids. Amino acids are shown on the x-axis indicated by single-letter code. In each graph, 91 scales’ amino acid hydrophobicity values are represented by black dots, their median is shown as a blue line with a shaded blue area representing the median absolute deviation. A dashed, horizontal line through zero is shown to guide the eye. The scale indicated by a subfigure title is highlighted in red for **(A)** the three scales with highest median feature importance and **(B)** the three scales with lowest median feature importance as determined in the learning experiment. The normalized scales’ sign was changed so that hydrophobicity of aspartic acid is always negative.

To retrieve information related to the hydrophobicity of individual amino acids, it is valuable to analyze hydrophobicity values of the normalized best and worst scales compared to the median of all hydrophobicity scales. If a particular scale performs better in feature selection than average, this can probably be attributed to the fact that the hydrophobicity values of certain amino acids are different from the median value. Figure 7 shows normalized hydrophobicity scale values for all amino acids for (Figure 7A) the three best and (Figure 7B) the three worst scales in the scope of this study. In the following, the hydrophobicity values of the highlighted scales are discussed in reference to the individual amino acid median hydrophobicity value and its MAD (of all 91 hydrophobicity scales). The three best scales are SET3, VHEG790101, and EISEN, in descending order. They have in common that arginine (single-letter code R, as indicated in Figure 7) has a significantly lower hydrophobicity value than in most other scales. This value falls well below the MAD range of the hydrophobicity value of all scales for arginine. Alanine (A) and glycine (G) are attributed a slightly larger hydrophobicity value than within the MAD of their distribution. SET3 and VHEG790101 also rate hydrophobicity of aspartic acid (D) lower and asparagine (N) higher compared to the MAD range. Compared to the median and the other two scales, SET3 has a markedly low hydrophobicity value for phenylalanine (F).

The three worst scales are NADH010107, ZIMJ680101, and WILM950103reverse, in descending order. For the worst scales, it is more difficult to identify patterns in the deviation from the median hydrophobicity values. However, a general trend in the worst three scales is that hydrophobicity values of hydrophobic amino acids are particularly low while charged amino acids are about average or above. Overall, the consensus of the best scales is that arginine should be attributed a lower hydrophobicity value than the population of scales would suggest, while alanine should be more hydrophobic than in most of the scales. Other charged amino acids are partly rated slightly less hydrophobic, such as aspartic acid and lysine. The worst scales’ accuracies are probably lower since a number of hydrophobic amino acids’ hydrophobicity values are comparably low, while, compared to the population of hydrophobicity scales, a number of charged or polar amino acids’ hydrophobicity values are relatively high. Amino acids such as cysteine or phenylalanine show conflicting trends in the worst and best scales and thus no conclusion thereof can be drawn.

The above analysis suggests that a unique property of arginine might contribute to its special scale position having both absolute lowest and relatively low hydrophobicity in the three best scales. Arginine’s role as an agent to reduce protein-protein interactions and increase solubility is only partially understood (Arakawa et al., 2007). As free amino acid in high concentrations (1 M), it has been shown to interact favorably with almost all amino acid side chains and peptide bonds. This means, that arginine can reduce both hydrophobic and electrostatic interactions (Arakawa et al., 2007). As an additive, arginine favorably interacts with tryptophan and therefore can suppress hydrophobic interactions leading to aggregation (Tsumoto et al., 2004). Exactly this effect is thought to bear the potential of introducing protein-protein interactions when arginine is present in abundance in the amino acid sequence of a protein (Warwicker et al., 2014). This conclusion was drawn by investigating the ratio of lysine to arginine (K/R) to highlight the specific effects of arginine on protein solubility as compared to lysine, since both bear one positive charge. It has also been shown that decreasing arginine content could increase solubility of a single-chain variable fragment (Austerberry et al., 2019). A negative arginine-related solubility effect was also seen in a study on a large data set of *E. coli* expressed proteins (Price et al., 2011). In this study on cVLPs, higher arginine content leads to decreased hydrophobicity values, which in turn leads to higher probability for soluble classification. This effect was observed although the K/R ratio [*mean* (*K*/*R*) = 0.32] was strongly unfavorable considering the results of Warwicker and colleagues (Warwicker et al., 2014). Another study showed that mutations from surface lysines to arginine in GFP could enhance its chemical stability (Sokalingam et al., 2012). However, protein folding was found to be aggravated. Protein solubility is a very complex topic and depends on a variety of factors of different dimensions, which is illustrated by the cVLP solubility problem. HBcAg dimers have low solubility in physiological pH and ionic strength, since hydrophobic and other interactions strongly favor VLPs (Ceres and Zlotnick, 2002). The assembly is an entropy-driven mechanism and is therefore similar to protein aggregation (Ceres and Zlotnick, 2002; Gorbenko and Trusova, 2011). Association relies on weak protein-protein interactions of HBcAg, such as hydrophobic interaction in a tyrosine pocket (Bourne et al., 2009) in the base of the molecule. This ordered aggregation is probably mandatory for the soluble state of HBcAg at the high expression levels in *E. coli*’s cytosol, since HBcAg dimers were found to be aggressively aggregating and forming capsids already at low concentrations (Ceres and Zlotnick, 2002). Protein insolubility during expression can therefore exhibit an entirely different origin than for other proteins – the association of the HBcAg proteins to structures that are not VLPs but unordered aggregates, which themselves are not soluble. Truncated wild-type HBcAg (Cp_1__–__149_, based on UniprotID: P03147; The UniProt Consortium, 2018) contains eight arginine residues with a K/R of 0.25, even though the arginine-rich C-terminus of the full-length HBcAg is not considered. To investigate this relationship with cVLPs, a one-level decision tree solely based on K/R ratio of all 568 observations was constructed, resulting in an inverse relationship as observed by Warwicker and colleagues (Warwicker et al., 2014). With the VLP solubility data, K/R values below the cut point are predicted as soluble with an Accuracy of 0.65 and MCC of 0.3, while in Warwicker’s study lower K/R lead to higher chances of insolubility. Arginine-based interactions could therefore be hypothesized to be of great importance in the recruitment of other HBcAg molecules to form VLPs eventually. Therefore, arginine’s property to increase protein-protein interactions when present in the amino acid sequences can be assumed to enhance VLP assembly, which is mandatory for significant levels of soluble HBcAg. Following this reasoning, substitution of arginine with lysine would maintain overall protein charge but probably promote the existence of either soluble HBcAg dimers incapable of assembly or insoluble HBcAg aggregates.

The role of arginine can also be discussed with respect to other amino acids. Tryptophan is not present in abundance in truncated wild-type HBcAg. Four tryptophan residues build the core of the HBcAg helices and are paired with either tyrosine, phenylalanine, or arginine residues (see also Supplementary Figure S2). Since arginine-tryptophan interactions were found to be extraordinarily strong (Arakawa et al., 2007), additional tryptophans in the epitope may result in misfolding during protein expression, since abundant arginines and other residues interact favorably with the tryptophans in the epitope region. This would give reason to the low feature importance observed in the three worst scales, which underrate tryptophan hydrophobicity. In the data set, 0, 1, or 2 tryptophans are introduced compared to the wild-type Cp_1__–__149_. Interestingly, observations containing two additional tryptophans are all insoluble, while observations containing zero or one additional tryptophan are found both in the soluble and in the insoluble group. As discussed above, valine probably also plays a vital role that is shown by its low hydrophobicity values in the worst scales. Arginine-valine interaction was found to be the only unfavorable interaction in single amino acid experiments (Arakawa et al., 2007). With the above reasoning, this could hamper assembly of HBcAg VLPs and therefore decrease VLP solubility.

### Systematic Classification Errors Based on Insertion Strategies

**FIGURE 8:**
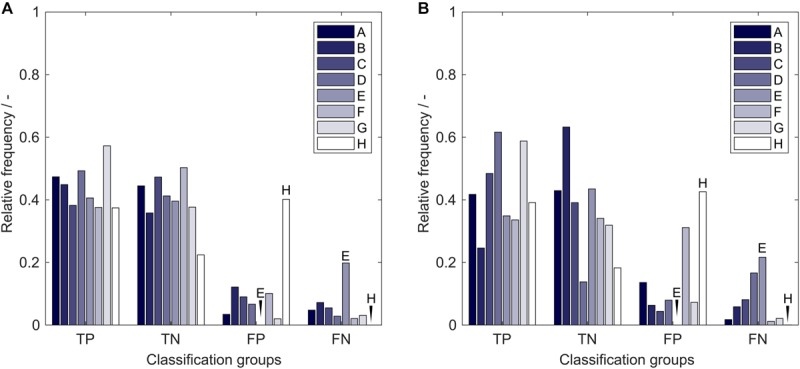
Relative frequency of classification groups based on insertion strategies A-H in **(A)** the training set and **(B)** the external test set of the 17,920 models in the learning experiment. Strategy E and H are marked additionally to guide the eye. TP, true positive; TN, true negative; FP, false positive; FN, false negative.

The average model performance with respect to the eight insertion strategies is related to properties of the utilized molecules that are strongly associated with the presented problem. If a strategy has a significantly higher relative frequency of FP classifications than FN classifications, the sEVC model systematically overestimates the solubility of observations created with this strategy. This indicates a particularly bad performance of this strategy. From Figure 8, it can be seen that this is the case for strategy H, both in training (A) and external test sets (B). During model construction, it would therefore be interesting to tweak strategy H’s solubility prediction so that the numbers of strategy H’s *FP* = *FN*. This can of course only be done for constructs where there is already a significant influence visible in the training set and when the training set is large enough. If a strategy is more numerous in the FN than in the FP group, the opposite case is true, where the model underestimates its solubility. These strategies are systematically good for solubility with respect to the model. This can, for example, be observed for strategy E. Its solubility prediction could be tweaked to higher solubility during model training. From the insertion sites and deletions that are different in the eight strategies we could, however, not find a relation that would explain the above-mentioned behavior. This relationship could be related to 3-D properties that cannot be explained by the 2-D amino acid sequence information only. The same approach has been tested on the 71 inserts, where no significant effects could be observed on the comparably large amount of different inserts and therefore their rarer occurrence (data not shown). The presented model performance analysis, which is based on the particular training set structure, can be used as a potent tool to learn about the characteristics of the data set and to boost model performance.

## Conclusion and Outlook

In this article, we presented a novel solubility prediction framework based on experimental and theoretical hydrophobicity scales that was applied to the prediction of chimeric HBcAg VLP solubility. In summary, little information was fed into our model, i.e., publicly available sequences, hydrophobicity scales, and solubility data.

The best models predicted with an MCC of > 0.6 on the external test set. Stratified training set sampling based on information on the inserted peptide sequence and the insertion strategy proved beneficial especially for small training set sizes. Evaluation of the contingency matrix revealed that certain epitope insertion strategies were overrepresented in the FP or the FN group of both training and test set and were therefore particularly limiting or promoting, respectively, for cVLP solubility. Detailed assessment of the best and worst features, i.e., hydrophobicity scales, suggested a special role for arginine for soluble cVLP expression. Contrary to reports on the solubility of other proteins, a large arginine content did not disrupt but rather improved cVLP solubility. We hypothesized that arginine’s positive interaction with almost all amino acids plays a crucial role in recruiting HBcAg dimers or larger building blocks to form a capsid, which in turn is required for meaningful levels of HBcAg concentrations in physiological buffers.

The presented framework proved to be applicable to small and larger training set sizes and could, with minor adaptations, be transferred to the prediction of monoclonal antibody solubility or even other biophysical properties. In the future, an informed design of scales that are orthogonal could greatly benefit the presented approach, as it would diversify the classifiers’ performances and therefore benefit the ensemble classifier. Additionally, it would be interesting to evaluate the model as a regression tool, avoiding the discretization that is performed during the sEVC procedure. Our results also suggest that building a global solubility model for all proteins is highly challenging and may only be feasible if a balanced data set of equally represented protein classes at very high observation numbers is available.

## Data Availability Statement

The raw data supporting the conclusions of this article will be made available by the authors, without undue reservation, to any qualified researcher. The amino acid sequence data for this article cannot be made available because they are confidential industry data.

## Author Contributions

JH initiated and supervised the work and edited the manuscript. TK provided the solubility data and was involved in the generation of the idea behind this manuscript. PV evolved the solubility prediction approach presented in this manuscript, performed the computational work and statistical analysis, and drafted the manuscript. PV, TK, and JH read and approved the final manuscript.

## Conflict of Interest

TK was employed by the company BioNTech SE.

The remaining authors declare that the research was conducted in the absence of any commercial or financial relationships that could be construed as a potential conflict of interest.

## Abbreviations

cVLP: chimeric virus-like particle
FN: false negative
FP: false positive
HBcAg: hepatitis B virus core antigen
MAD: median absolute deviation
MC-CV: Monte Carlo cross-validation
MCC: Matthew’s correlation coefficient
MIR: major immunodominant region
PCA: principal component analysis
sEVC: soft ensemble vote classifier
SVM: support vector machine
TN: True negative
TP: true positive
VLP: virus-like particle.
