# Supplementary material for: Ensembles of Hydrophobicity Scales as Potent Classifiers for Chimeric Virus-Like Particle Solubility – An Amino Acid Sequence-Based Machine Learning Approach
Source: Front Bioeng Biotechnol. 2020 May 5;8:395. doi: 10.3389/fbioe.2020.00395 (PMC7217080; doi:10.3389/fbioe.2020.00395)
Supplement: Supplementary file 1 [file Data_Sheet_1.DOCX]

Supplementary Material

# Normalized Hydrophobicity Scales

Table S1 shows normalized hydrophobicity scales that were used in this study. Scales were derived from a recent study on hydrophobicity scales for peptide classification (Simm et al. 2016). Reversed scales were excluded if there was a complementary, non-reversed scale available, and the remaining 91 scales were centered and scaled to unit variance. The scale IDs (feature names) were adapted from above-mentioned study removing spaces. For reference to the original publications of the hydrophobicity scales we refer to their article.

# Comparison of MCC and accuracy

To compare the presented data with available models, we must understand the relation between the MCC and the typically reported accuracy. In the ideal balanced case, where $TP=TN$ and $FP=FN$ (implying $n_{total,positive}=n_{total,negative}$ which is a balanced data set), the relationship between accuracy $A$and the MCC is

$$\begin{aligned} A=0.5+0.5\times MCC.\#\left( S1 \right) \end{aligned}$$

When $TP\neq TN,$ it is

$$\begin{aligned} A<0.5+0.5\times MCC.\#\left( S2 \right) \end{aligned}$$

Contrary to this, class imbalance in $FP$ and $FN$ increases MCC. It is however less pronounced in models that predict better than average random. Reported accuracy of other solubility models varies greatly and falls into the region of $.62 - .83$ (Idicula-Thomas et al. 2006; Smialowski et al. 2006; Magnan, Randall, and Baldi 2009; Hebditch et al. 2017). Assuming the ideal balanced case, this translates to an MCC analogue of $0.24-0.66$. It is important to note, that this is the ideal case and therefore typically results in overestimated MCCs. Compared to these values, the best models in the learning experiment are close to the best reported accuracies of previous models. Class imbalance would be favorable to the presented model, as the assumed MCC of previous models is overestimated, while it is included in the actual MCC in this article.

# Feature importance

In the learning experiment, 17290 models were created with varying training set size and number of included decision trees within the soft ensemble vote classifier. The median feature importance and the median absolute deviation (MAD) of the features were computed and are shown in Table S2.

**Table S1:** Centered and unit-variance scaled hydrophobicity scales derived from Simm *et al.* (2016) (Simm et al. 2016). Reversed scales were excluded if there was a complementary, non-reversed scale available, resulting in 91 scales. Each amino acid, represented in single-letter code, is assigned a hydrophobicity value by each hydrophobicity scale.

| Scale-ID/Amino Acid | A | R | N | D | C | Q | E | G | H | I | L | K | M | F | P | S | T | W | Y | V |
| --- | --- | --- | --- | --- | --- | --- | --- | --- | --- | --- | --- | --- | --- | --- | --- | --- | --- | --- | --- | --- |
| CIDH920101 | -0,611 | -0,406 | -0,367 | -1,654 | 0,598 | -1,137 | -0,952 | -1,147 | 0,871 | 0,569 | 1,086 | -0,523 | 1,164 | 1,271 | -0,289 | -1,127 | -0,854 | 1,173 | 1,280 | 1,056 |
| CIDH920105 | -0,117 | -0,548 | -0,891 | -1,155 | 0,617 | -1,214 | -1,253 | -0,920 | 0,118 | 1,636 | 0,980 | -0,538 | 0,843 | 1,185 | -0,225 | -1,086 | -0,891 | 1,538 | 0,950 | 0,970 |
| ESID840101 | 0,623 | -2,534 | -0,775 | -0,901 | 0,293 | -0,854 | -0,744 | 0,481 | -0,398 | 1,376 | 1,062 | -1,497 | 0,638 | 1,188 | 0,120 | -0,178 | -0,053 | 0,811 | 0,261 | 1,078 |
| MANP780101 | 0,060 | -0,733 | -0,924 | -1,285 | 1,114 | -0,708 | -0,625 | -0,282 | -0,454 | 1,774 | 1,285 | -0,962 | 0,962 | 0,714 | -0,955 | -1,044 | -0,752 | 0,670 | 0,346 | 1,800 |
| PONP800101 | -0,177 | -0,713 | -1,045 | -1,066 | 1,620 | -0,855 | -0,917 | -0,360 | 0,202 | 1,511 | 1,057 | -1,181 | 1,213 | 0,603 | -0,917 | -0,869 | -0,605 | 0,277 | 0,508 | 1,715 |
| PONP800102 | -0,172 | -0,688 | -1,096 | -1,090 | 1,938 | -0,777 | -0,962 | -0,369 | -0,025 | 1,339 | 0,944 | -1,383 | 1,237 | 0,701 | -0,796 | -0,612 | -0,516 | 0,332 | 0,408 | 1,587 |
| PONP800103 | -0,158 | -0,667 | -1,176 | -1,119 | 1,905 | -0,667 | -1,063 | -0,384 | -0,328 | 1,113 | 0,831 | -1,600 | 1,396 | 0,944 | -0,639 | -0,243 | -0,384 | 0,463 | 0,294 | 1,481 |
| PONP800104 | 0,699 | -1,129 | -0,388 | -1,360 | 1,347 | -1,113 | -0,149 | 2,018 | -0,889 | 1,455 | 0,977 | -0,604 | 0,506 | 1,031 | -0,951 | -1,144 | 0,198 | -0,527 | -0,080 | 0,105 |
| PONP800105 | 0,244 | -0,802 | -1,917 | -0,387 | 1,244 | -1,740 | -0,533 | -0,079 | 0,821 | -0,140 | 1,698 | -0,771 | 1,498 | -0,079 | -0,140 | -0,710 | 0,167 | -0,294 | 0,367 | 1,552 |
| PONP800106 | -0,821 | -0,553 | -0,694 | -1,145 | 1,631 | -0,088 | -0,088 | -0,624 | 0,165 | 0,785 | 0,870 | -1,343 | 2,230 | 1,011 | -0,856 | -0,462 | -0,920 | -0,300 | -0,222 | 1,426 |
| PRAM900101reverse | 0,607 | -2,235 | -0,701 | -1,600 | 0,690 | -0,560 | -1,395 | 0,485 | -0,335 | 0,915 | 0,851 | -1,517 | 0,973 | 1,037 | 0,241 | 0,402 | 0,524 | 0,666 | 0,138 | 0,812 |
| SWER830101 | -0,401 | -0,591 | -0,921 | -1,311 | 0,170 | -0,911 | -1,221 | -0,671 | -0,641 | 1,250 | 1,220 | -0,671 | 1,020 | 1,920 | -0,491 | -0,551 | -0,281 | 0,500 | 1,670 | 0,910 |
| NADH010101 | 0,716 | -1,875 | -0,900 | -0,943 | 1,337 | -1,393 | -1,307 | -0,022 | -0,686 | 1,241 | 1,112 | -0,162 | 0,930 | 1,080 | -0,751 | -0,269 | 0,020 | 0,727 | -0,022 | 1,166 |
| NADH010102 | 0,590 | -1,354 | -0,756 | -0,696 | 1,448 | -1,195 | -1,065 | -0,048 | -0,467 | 1,139 | 1,109 | -1,963 | 0,810 | 1,159 | -0,706 | -0,178 | 0,052 | 0,770 | 0,191 | 1,159 |
| NADH010103 | 0,357 | -1,240 | -0,867 | -0,580 | 1,720 | -1,187 | -1,038 | -0,271 | -0,452 | 1,028 | 1,017 | -1,655 | 0,741 | 1,284 | -0,942 | -0,409 | 0,027 | 1,007 | 0,304 | 1,156 |
| NADH010104 | 0,196 | -1,190 | -0,949 | -0,469 | 1,834 | -1,190 | -0,960 | -0,393 | -0,426 | 1,004 | 0,982 | -1,506 | 0,742 | 1,288 | -1,048 | -0,524 | 0,065 | 1,135 | 0,327 | 1,080 |
| NADH010106 | -0,232 | -0,946 | -1,176 | 0,228 | 2,288 | -1,061 | -0,382 | -0,831 | -0,865 | 0,665 | 0,654 | -0,727 | 0,665 | 1,057 | -1,475 | -0,762 | 0,619 | 1,206 | 0,021 | 1,057 |
| NADH010107 | -0,434 | -0,754 | -1,213 | 1,617 | 2,281 | -0,721 | 0,542 | -0,959 | -0,992 | -0,188 | -0,122 | 0,526 | 0,091 | 0,567 | -1,500 | -0,844 | 1,010 | 1,051 | -0,475 | 0,518 |
| WILM950101 | -0,390 | -0,910 | -0,281 | -0,538 | -0,143 | -0,246 | -0,481 | -0,304 | -1,706 | 1,568 | 1,579 | -1,351 | -0,304 | 2,324 | -0,017 | -0,779 | -0,052 | 0,887 | 0,658 | 0,486 |
| WILM950102 | 0,475 | 0,069 | -0,686 | -1,155 | -0,089 | -0,811 | -0,441 | -0,650 | -0,528 | 1,001 | 1,655 | -1,137 | -1,238 | 2,422 | -0,343 | -0,722 | 0,233 | 1,457 | 0,108 | 0,380 |
| WILM950103reverse | 1,008 | 1,541 | 0,206 | 0,248 | -2,545 | 0,488 | 0,092 | 1,076 | -1,853 | -0,505 | 0,206 | 1,242 | -0,908 | 0,917 | -0,538 | -0,041 | -0,275 | -0,372 | -0,294 | 0,307 |
| WILM950104 | -1,374 | 0,196 | 0,678 | -0,633 | 1,563 | -0,378 | 0,077 | -0,864 | -1,637 | 2,452 | -0,007 | 0,180 | -0,194 | 0,583 | -0,501 | 0,328 | -0,366 | 0,989 | -1,470 | 0,379 |
| KUHL950101reverse | 0,350 | -2,003 | -0,885 | -1,326 | 1,026 | -0,856 | -1,621 | 0,644 | -0,268 | 1,262 | 0,997 | -0,591 | 0,703 | 1,262 | 0,615 | -0,297 | -0,444 | 0,585 | -0,297 | 1,144 |
| JURD980101 | 0,547 | -1,503 | -0,974 | -1,007 | 1,010 | -1,033 | -0,874 | -0,028 | -0,874 | 1,671 | 1,439 | -1,175 | 0,811 | 1,109 | -0,445 | 0,018 | -0,048 | 0,031 | -0,246 | 1,572 |
| KIDA850101reverse | 0,263 | -1,822 | -0,789 | -0,789 | 1,023 | -1,072 | -1,140 | 0,156 | -0,273 | 0,750 | 1,072 | -1,656 | 0,711 | 1,393 | 0,731 | -0,409 | -0,614 | 1,530 | 0,546 | 0,390 |
| ENGD860101reverse | 0,607 | -2,234 | -0,701 | -1,600 | 0,689 | -0,558 | -1,396 | 0,484 | -0,333 | 0,914 | 0,852 | -1,519 | 0,975 | 1,036 | 0,239 | 0,403 | 0,525 | 0,668 | 0,137 | 0,811 |
| KYTJ820101 | 0,767 | -1,342 | -1,008 | -1,008 | 1,001 | -1,008 | -1,008 | 0,030 | -0,907 | 1,671 | 1,436 | -1,142 | 0,800 | 1,101 | -0,372 | -0,104 | -0,070 | -0,137 | -0,271 | 1,570 |
| ENGEL | 0,607 | -2,234 | -0,701 | -1,600 | 0,689 | -0,558 | -1,396 | 0,484 | -0,333 | 0,914 | 0,852 | -1,519 | 0,975 | 1,036 | 0,239 | 0,403 | 0,525 | 0,668 | 0,137 | 0,811 |
| JONES | -0,468 | -0,485 | -1,131 | -0,647 | 0,087 | -1,209 | -0,637 | -1,124 | -0,468 | 1,478 | 0,642 | 0,189 | 0,216 | 1,238 | 1,153 | -1,148 | -1,148 | 2,006 | 1,069 | 0,385 |
| CIDBB | -0,358 | -0,368 | -0,963 | -0,973 | 0,462 | -0,671 | -1,559 | -1,100 | 0,140 | 1,077 | 0,930 | -0,368 | 0,960 | 1,213 | -0,290 | -1,188 | -0,856 | 1,916 | 1,213 | 0,784 |
| CIDA | 0,233 | -0,623 | -0,993 | -1,178 | 0,564 | -1,139 | -0,925 | -0,915 | -0,273 | 1,994 | 1,031 | -0,662 | 1,060 | 0,865 | -0,176 | -0,701 | -1,285 | 1,157 | 0,904 | 1,060 |
| CIDAB | 0,090 | -0,759 | -0,954 | -1,100 | 1,134 | -1,247 | -1,237 | -0,632 | -0,320 | 1,934 | 0,860 | -0,681 | 0,509 | 1,182 | -0,281 | -0,886 | -0,681 | 1,397 | 0,568 | 1,104 |
| PONG1 | 0,204 | -0,283 | -0,792 | -1,257 | 1,141 | -0,747 | -1,025 | -0,433 | -0,268 | 1,920 | 1,058 | -1,324 | 0,631 | 0,833 | -1,287 | -0,822 | -0,493 | 0,886 | 0,594 | 1,463 |
| PONG2 | 0,376 | -0,253 | 0,124 | -1,498 | 1,695 | -0,527 | -2,068 | 0,243 | 0,791 | 0,799 | -0,061 | -1,476 | -0,039 | -0,135 | -0,379 | 0,710 | -0,646 | 2,117 | 0,036 | 0,191 |
| PONG3 | 0,321 | -0,300 | -0,366 | -1,534 | 1,580 | -0,706 | -1,724 | -0,101 | 0,297 | 1,506 | 0,553 | -1,559 | 0,330 | 0,388 | -0,921 | -0,060 | -0,631 | 1,671 | 0,346 | 0,909 |
| KIDER | 0,263 | -1,822 | -0,789 | -0,789 | 1,023 | -1,072 | -1,140 | 0,156 | -0,273 | 0,750 | 1,072 | -1,656 | 0,711 | 1,393 | 0,731 | -0,409 | -0,614 | 1,530 | 0,546 | 0,390 |
| WOLR790101 | 1,119 | -2,548 | -0,830 | -0,830 | 0,589 | -0,780 | -0,920 | 1,198 | -0,930 | 1,159 | 1,178 | -0,800 | 0,549 | 0,669 | 0,539 | -0,050 | -0,020 | -0,190 | -0,230 | 1,129 |
| CHOTA | -1,264 | 1,253 | -0,235 | -0,463 | -0,807 | 0,223 | 0,452 | -2,180 | 0,566 | 0,109 | -0,006 | 0,681 | 0,338 | 0,910 | -0,578 | -1,264 | -0,692 | 1,940 | 1,367 | -0,349 |
| ROSEB | -1,107 | 0,683 | -0,726 | -0,845 | -0,033 | -0,344 | -0,463 | -1,680 | 0,539 | 0,587 | 0,730 | -0,415 | 0,945 | 1,446 | -0,964 | -1,131 | -0,654 | 2,186 | 1,064 | 0,181 |
| ROSEAreverse | 1,385 | -1,608 | 0,344 | 0,496 | 0,777 | -0,242 | -0,090 | 2,035 | -0,437 | 0,018 | -0,242 | -0,957 | -0,459 | -0,892 | 0,756 | 1,124 | 0,647 | -1,825 | -1,196 | 0,365 |
| COHEN | -0,874 | -0,665 | -1,607 | -1,189 | -0,456 | -0,874 | -0,665 | -1,189 | 0,382 | 0,696 | 0,801 | 0,487 | 1,534 | 1,220 | 1,115 | -0,979 | -0,246 | 1,744 | 0,487 | 0,278 |
| JACWH | 0,626 | -3,164 | -0,379 | -1,099 | 0,687 | -0,325 | -0,922 | 0,565 | -0,501 | 0,755 | 0,782 | -1,214 | 0,803 | 0,844 | 0,368 | 0,232 | 0,273 | 0,497 | 0,450 | 0,721 |
| CASSI | 0,198 | -0,671 | -0,478 | -1,347 | 1,839 | -1,057 | -1,250 | -0,092 | 0,391 | 1,356 | 0,487 | -1,540 | 0,487 | 0,970 | -0,960 | -0,671 | -0,381 | 1,549 | 0,487 | 0,681 |
| MDK0 | 0,673 | -1,499 | -0,973 | -1,006 | 1,002 | -1,032 | -0,874 | -0,097 | -0,874 | 1,660 | 1,430 | -1,173 | 0,804 | 1,101 | -0,446 | 0,015 | -0,051 | 0,028 | -0,249 | 1,561 |
| MDK1 | 0,746 | -1,581 | -0,949 | -0,949 | 0,846 | -1,248 | -1,049 | 0,081 | -0,750 | 1,710 | 1,378 | -1,182 | 0,846 | 1,046 | -0,317 | -0,052 | 0,115 | 0,115 | -0,218 | 1,411 |
| BULDG | 0,737 | 0,823 | 1,037 | 0,737 | 0,468 | 1,123 | 0,629 | 0,951 | 0,823 | -1,475 | -1,690 | 0,576 | -0,627 | -1,551 | -0,101 | 0,533 | 0,393 | -1,207 | -1,454 | -0,724 |
| GUYH850101 | 0,175 | 1,832 | 0,523 | 0,798 | -1,216 | 0,953 | 0,844 | 0,386 | -0,374 | -0,951 | -0,996 | 1,365 | -1,372 | -1,857 | 0,752 | 0,560 | 0,148 | -0,383 | -0,108 | -1,079 |
| MIYS850101 | -0,166 | -0,704 | -0,923 | -0,979 | 1,050 | -0,849 | -0,951 | -0,563 | -0,274 | 1,470 | 1,302 | -1,279 | 1,527 | 1,564 | -0,849 | -0,746 | -0,559 | 0,923 | 0,096 | 0,909 |
| Wilson | -0,631 | -0,848 | -0,604 | -0,929 | 1,157 | -0,604 | -0,550 | -0,225 | -0,902 | 0,615 | 1,238 | -1,525 | 0,127 | 1,482 | 0,046 | -0,712 | -1,146 | 1,590 | 1,373 | 1,048 |
| CHOC760103 | 0,586 | -1,490 | -0,873 | -0,704 | 1,260 | -1,153 | -0,536 | 0,474 | -0,592 | 1,821 | 0,979 | -1,378 | 0,699 | 1,260 | -0,536 | -0,311 | -0,255 | -0,031 | -0,704 | 1,484 |
| EISEN | 0,620 | -2,531 | -0,780 | -0,900 | 0,290 | -0,850 | -0,740 | 0,480 | -0,400 | 1,380 | 1,060 | -1,500 | 0,640 | 1,190 | 0,120 | -0,180 | -0,050 | 0,810 | 0,260 | 1,080 |
| JANJ790102 | 0,589 | -1,824 | -0,547 | -0,689 | 1,441 | -0,831 | -0,831 | 0,589 | 0,021 | 1,157 | 0,873 | -2,392 | 0,731 | 0,873 | -0,263 | 0,021 | -0,121 | 0,589 | -0,405 | 1,015 |
| RaoArgos | 0,954 | -1,390 | -1,041 | -1,467 | 0,780 | -1,041 | -1,196 | 0,431 | -0,363 | 1,109 | 1,167 | -1,506 | 1,070 | 1,361 | -0,634 | 0,199 | 0,412 | 0,257 | -0,073 | 0,973 |
| NOZY710101Tanford | 0,491 | -2,485 | -0,832 | -0,180 | 0,180 | -0,898 | -0,794 | 0,359 | -0,472 | 1,209 | 1,351 | -1,512 | 0,510 | 1,030 | 0,019 | -0,265 | -0,142 | 0,671 | 0,151 | 1,606 |
| Welling | 0,946 | 0,585 | -0,270 | 0,629 | -0,542 | 0,148 | -0,232 | -0,947 | 2,193 | -1,631 | 0,693 | 1,522 | -2,219 | -0,675 | -0,118 | 0,053 | -0,067 | -0,504 | 0,300 | 0,135 |
| PARJ860101 | 0,177 | 0,508 | 0,951 | 1,424 | 0,066 | 0,793 | 1,077 | 0,745 | 0,177 | -1,418 | -1,608 | 0,745 | -0,818 | -1,608 | 0,177 | 0,872 | 0,666 | -1,734 | -0,455 | -0,739 |
| ROSG850102 | 0,030 | -0,837 | -0,924 | -1,010 | 1,505 | -1,010 | -1,010 | -0,143 | 0,377 | 1,244 | 0,984 | -1,877 | 0,984 | 1,244 | -0,837 | -0,663 | -0,317 | 0,984 | 0,204 | 1,071 |
| Bishop | 0,200 | 0,321 | 0,442 | 1,411 | -0,406 | 0,684 | 2,381 | -0,042 | 0,200 | -0,406 | -0,769 | 0,563 | -0,285 | -1,375 | 0,442 | 0,079 | 0,079 | -2,344 | -1,254 | 0,079 |
| Wimleyreverse | -0,372 | -1,499 | -0,673 | -0,312 | 0,075 | -0,604 | -0,037 | -0,931 | -0,037 | 1,021 | 1,133 | -2,350 | 0,634 | 1,529 | -0,062 | -0,338 | -0,157 | 1,855 | 0,669 | 0,454 |
| Wimley | -0,086 | -0,832 | -0,377 | -1,322 | 0,393 | -0,564 | -2,244 | 0,101 | -0,086 | 0,474 | 0,766 | -1,042 | 0,381 | 1,431 | -0,412 | -0,039 | -0,051 | 2,271 | 1,209 | 0,031 |
| ARGP820101 | -0,469 | -0,481 | -1,135 | -0,651 | 0,088 | -1,208 | -0,639 | -1,123 | -0,469 | 1,480 | 0,645 | 0,185 | 0,221 | 1,238 | 1,153 | -1,147 | -1,147 | 2,001 | 1,068 | 0,390 |
| FAUJ830101 | -0,167 | -1,449 | -1,051 | -1,216 | 1,027 | -0,682 | -1,089 | -0,468 | -0,342 | 1,280 | 1,183 | -1,429 | 0,726 | 1,270 | 0,231 | -0,507 | -0,216 | 1,717 | 0,464 | 0,717 |
| JOND750101 | -0,467 | -0,484 | -1,132 | -0,646 | 0,087 | -1,209 | -0,637 | -1,123 | -0,467 | 1,477 | 0,642 | 0,190 | 0,215 | 1,239 | 1,153 | -1,149 | -1,149 | 2,006 | 1,068 | 0,386 |
| LEVM760101reverse | 0,089 | -1,795 | -0,288 | -1,526 | 0,358 | -0,288 | -1,526 | -0,180 | 0,089 | 0,789 | 0,789 | -1,795 | 0,519 | 1,165 | 0,573 | -0,342 | 0,035 | 1,650 | 1,058 | 0,627 |
| ZIMJ680101 | -0,427 | -0,427 | -1,137 | -0,609 | 0,196 | -1,223 | -0,600 | -1,127 | -0,168 | 1,720 | 1,193 | 0,311 | 0,119 | 1,414 | 1,366 | -1,089 | -0,705 | -0,926 | 1,625 | 0,493 |
| NADH010105 | 0,012 | -1,206 | -1,171 | -0,272 | 2,022 | -1,301 | -0,946 | -0,603 | -0,639 | 0,934 | 0,946 | -0,378 | 0,792 | 1,277 | -1,277 | -0,698 | 0,130 | 1,100 | 0,237 | 1,041 |
| PONP930101 | 0,204 | -0,283 | -0,792 | -1,257 | 1,141 | -0,747 | -1,025 | -0,433 | -0,268 | 1,920 | 1,058 | -1,324 | 0,631 | 0,833 | -1,287 | -0,822 | -0,493 | 0,886 | 0,594 | 1,463 |
| COWR900101 | 0,238 | -1,310 | -0,896 | -0,489 | 0,566 | -0,841 | -0,380 | -0,091 | -1,873 | 1,324 | 1,317 | -1,678 | 0,832 | 1,270 | 0,582 | -0,591 | -0,294 | 1,051 | 0,308 | 0,957 |
| BLAS910101 | 0,276 | -1,563 | -0,851 | -1,474 | 0,454 | -0,821 | -1,444 | -0,080 | -1,059 | 1,225 | 1,225 | -0,732 | 0,632 | 1,403 | 0,543 | -0,495 | -0,228 | 1,047 | 1,047 | 0,898 |
| FASG890101 | 0,232 | 1,030 | 0,634 | 0,772 | -1,772 | 0,827 | 1,095 | 0,304 | -0,118 | -1,349 | -1,304 | 1,638 | -0,954 | -1,294 | 0,562 | 0,903 | 0,573 | -0,837 | -0,043 | -0,899 |
| FAUCH | -0,168 | -1,449 | -1,050 | -1,216 | 1,028 | -0,683 | -1,089 | -0,468 | -0,341 | 1,279 | 1,184 | -1,428 | 0,725 | 1,268 | 0,231 | -0,507 | -0,214 | 1,717 | 0,464 | 0,718 |
| PONNU | -0,176 | -0,712 | -1,046 | -1,066 | 1,621 | -0,855 | -0,916 | -0,360 | 0,202 | 1,512 | 1,058 | -1,182 | 1,212 | 0,601 | -0,916 | -0,868 | -0,606 | 0,277 | 0,506 | 1,713 |
| JANIN | 0,245 | -1,091 | -0,839 | -0,839 | 2,661 | -0,924 | -0,924 | 0,327 | -0,505 | 1,410 | 0,828 | -1,132 | 0,408 | 0,661 | -0,672 | -0,505 | -0,590 | 0,160 | 0,078 | 1,243 |
| GUYFE | -0,219 | -1,057 | -0,670 | -1,015 | 1,308 | -0,940 | -0,983 | -0,595 | 0,371 | 1,255 | 1,145 | -1,423 | 1,209 | 1,653 | -0,908 | -0,691 | -0,446 | 0,790 | 0,200 | 1,017 |
| CHOTH | 0,585 | -1,489 | -0,873 | -0,705 | 1,259 | -1,154 | -0,534 | 0,475 | -0,591 | 1,822 | 0,981 | -1,379 | 0,699 | 1,259 | -0,534 | -0,309 | -0,256 | -0,032 | -0,705 | 1,483 |
| VHEG790101 | 0,731 | -2,592 | -0,323 | -1,555 | -0,303 | -0,187 | -1,139 | 0,460 | -0,455 | 1,137 | 1,104 | -0,676 | 0,526 | 1,375 | -0,425 | 0,050 | 0,219 | 1,002 | 0,050 | 1,002 |
| ROSEM | 0,224 | -1,909 | -1,151 | -0,824 | 0,603 | -1,081 | -0,922 | -0,090 | -0,599 | 1,323 | 1,323 | -1,240 | 0,692 | 1,323 | 0,729 | -0,136 | 0,107 | 1,248 | -0,548 | 0,930 |
| LEVIT | 0,089 | -1,795 | -0,288 | -1,526 | 0,358 | -0,288 | -1,526 | -0,181 | 0,089 | 0,789 | 0,789 | -1,795 | 0,520 | 1,165 | 0,575 | -0,343 | 0,033 | 1,651 | 1,058 | 0,627 |
| GIBRA | -0,232 | -1,030 | -0,634 | -0,772 | 1,772 | -0,827 | -1,095 | -0,304 | 0,118 | 1,349 | 1,304 | -1,638 | 0,954 | 1,294 | -0,562 | -0,903 | -0,573 | 0,837 | 0,043 | 0,899 |
| ROSEF | 0,029 | -0,837 | -0,923 | -1,010 | 1,503 | -1,010 | -1,010 | -0,143 | 0,378 | 1,244 | 0,985 | -1,879 | 0,985 | 1,244 | -0,837 | -0,664 | -0,316 | 0,985 | 0,205 | 1,071 |
| SweetEisenberg | -0,183 | -1,822 | -0,528 | -0,800 | 1,191 | -0,952 | -0,843 | -0,392 | 0,358 | 0,929 | 1,234 | -1,360 | 1,294 | 1,825 | -0,753 | -0,564 | -0,156 | 0,368 | 0,096 | 1,055 |
| NNEIG | 0,162 | -0,284 | -0,891 | -1,448 | 1,854 | -0,828 | -1,383 | 0,057 | -0,143 | 1,549 | 0,825 | -1,501 | 0,582 | 0,733 | -0,979 | -0,586 | -0,156 | 0,762 | 0,378 | 1,297 |
| SWEET | -0,506 | -1,768 | -1,067 | -0,424 | 0,143 | -0,678 | -0,443 | -0,226 | -0,232 | 1,341 | 1,341 | -1,787 | 0,475 | 1,392 | 0,596 | -0,761 | -0,487 | 1,404 | 0,812 | 0,876 |
| WOLR810101reverse | 1,099 | -2,502 | -0,816 | -1,025 | 0,575 | -0,766 | -0,901 | 1,173 | -0,913 | 1,133 | 1,155 | -0,789 | 0,535 | 0,654 | 0,779 | -0,055 | -0,025 | -0,190 | -0,227 | 1,107 |
| CowanWhittacker | 0,240 | -1,196 | -0,800 | -1,701 | 0,558 | -0,754 | -1,546 | -0,032 | -0,536 | 1,389 | 1,366 | -1,227 | 0,822 | 1,280 | 0,620 | -0,521 | -0,241 | 1,016 | 0,271 | 0,993 |
| ROSM880101 | -0,600 | 2,139 | 1,094 | 1,414 | -0,529 | 0,914 | 1,120 | -0,456 | 0,363 | -1,104 | -1,104 | 0,858 | -0,735 | -1,151 | -0,832 | 0,477 | 0,372 | -1,070 | -0,246 | -0,924 |
| ROSM880102 | -0,203 | 1,895 | 1,150 | 0,828 | -0,815 | 1,081 | 0,924 | 0,105 | 0,607 | -1,285 | -1,285 | 1,237 | -0,663 | -1,386 | -0,700 | 0,151 | -0,088 | -1,211 | 0,556 | -0,898 |
| ROSM880103reverse | 0,641 | 0,947 | -0,885 | -0,580 | 0,336 | -0,275 | -2,107 | 1,863 | -1,191 | 0,641 | 0,031 | 0,641 | 0,947 | -0,275 | -0,885 | 0,641 | 0,641 | 0,031 | -1,801 | 0,641 |
| SET1 | 0,662 | -2,482 | -0,530 | -1,870 | 1,751 | -0,452 | -0,836 | 0,880 | 0,145 | 0,677 | 1,267 | -0,327 | 0,400 | -0,460 | -0,460 | 0,334 | 0,271 | -0,135 | 0,249 | 0,917 |
| SET2 | 0,850 | -1,912 | -1,430 | -0,956 | 0,830 | -0,511 | -1,306 | 0,682 | -0,093 | 1,075 | 1,260 | -1,121 | 0,959 | -0,740 | -0,740 | 0,666 | 0,220 | 0,509 | 0,541 | 1,216 |
| SET3 | 0,977 | -2,185 | -0,429 | -1,437 | 0,551 | -0,472 | -0,666 | 0,648 | -0,193 | 1,082 | 1,369 | -0,898 | 0,877 | -1,123 | -1,123 | 0,563 | 0,601 | 0,838 | -0,015 | 1,035 |

**Table S2:** Median and median absolute deviation (MAD) of feature importance (scale accuracy on training data) in the learning experiment. Each data point represents 19 different training set sizes each evaluated 910 times. Scales are sorted according to feature importance in descending order. Scale no. 1 has highest feature importance, scale no. 91 has lowest feature importance.

| No. | Median accuracy | MAD of accuracy | No. | Median accuracy | MAD of accuracy | No. | Median accuracy | MAD of accuracy | No. | Median accuracy | MAD of accuracy | No. | Median accuracy | MAD of accuracy |
| --- | --- | --- | --- | --- | --- | --- | --- | --- | --- | --- | --- | --- | --- | --- |
| 1 | 0.85 | 0.02 | **20** | 0.82 | 0.02 | **39** | 0.81 | 0.02 | **58** | 0.78 | 0.02 | **77** | 0.70 | 0.03 |
| 2 | 0.84 | 0.02 | **21** | 0.82 | 0.02 | **40** | 0.81 | 0.02 | **59** | 0.78 | 0.03 | **78** | 0.70 | 0.03 |
| 3 | 0.84 | 0.02 | **22** | 0.82 | 0.02 | **41** | 0.81 | 0.02 | **60** | 0.78 | 0.02 | **79** | 0.70 | 0.03 |
| 4 | 0.83 | 0.02 | **23** | 0.82 | 0.02 | **42** | 0.80 | 0.02 | **61** | 0.77 | 0.02 | **80** | 0.68 | 0.03 |
| 5 | 0.83 | 0.02 | **24** | 0.82 | 0.02 | **43** | 0.80 | 0.02 | **62** | 0.76 | 0.02 | **81** | 0.68 | 0.03 |
| 6 | 0.83 | 0.02 | **25** | 0.82 | 0.02 | **44** | 0.80 | 0.02 | **63** | 0.76 | 0.02 | **82** | 0.68 | 0.03 |
| 7 | 0.83 | 0.02 | **26** | 0.82 | 0.02 | **45** | 0.80 | 0.02 | **64** | 0.76 | 0.03 | **83** | 0.67 | 0.03 |
| 8 | 0.83 | 0.02 | **27** | 0.82 | 0.02 | **46** | 0.80 | 0.02 | **65** | 0.76 | 0.02 | **84** | 0.66 | 0.03 |
| 9 | 0.82 | 0.02 | **28** | 0.81 | 0.02 | **47** | 0.79 | 0.02 | **66** | 0.76 | 0.02 | **85** | 0.66 | 0.03 |
| 10 | 0.82 | 0.02 | **29** | 0.81 | 0.02 | **48** | 0.79 | 0.02 | **67** | 0.76 | 0.03 | **86** | 0.66 | 0.03 |
| 11 | 0.82 | 0.02 | **30** | 0.81 | 0.02 | **49** | 0.79 | 0.02 | **68** | 0.74 | 0.03 | **87** | 0.66 | 0.03 |
| 12 | 0.82 | 0.02 | **31** | 0.81 | 0.02 | **50** | 0.79 | 0.03 | **69** | 0.74 | 0.03 | **88** | 0.62 | 0.03 |
| 13 | 0.82 | 0.02 | **32** | 0.81 | 0.02 | **51** | 0.78 | 0.02 | **70** | 0.74 | 0.03 | **89** | 0.58 | 0.03 |
| 14 | 0.82 | 0.02 | **33** | 0.81 | 0.02 | **52** | 0.78 | 0.02 | **71** | 0.74 | 0.03 | **90** | 0.57 | 0.03 |
| 15 | 0.82 | 0.02 | **34** | 0.81 | 0.02 | **53** | 0.78 | 0.02 | **72** | 0.73 | 0.03 | **91** | 0.54 | 0.04 |
| 16 | 0.82 | 0.02 | **35** | 0.81 | 0.02 | **54** | 0.78 | 0.02 | **73** | 0.73 | 0.02 |  |  |  |
| 17 | 0.82 | 0.02 | **36** | 0.81 | 0.02 | **55** | 0.78 | 0.02 | **74** | 0.73 | 0.03 |  |  |  |
| 18 | 0.82 | 0.02 | **37** | 0.81 | 0.02 | **56** | 0.78 | 0.02 | **75** | 0.71 | 0.03 |  |  |  |
| 19 | 0.82 | 0.02 | **38** | 0.81 | 0.02 | **57** | 0.78 | 0.02 | **76** | 0.71 | 0.02 |  |  |  |

# Principal Component Analysis

The centered and unit-variance scaled hydrophobicity scales were analyzed by principal component analysis using MATLAB. Figure S1 shows the explained variance per principal component and as cumulative explained variance over principal components. 68.8% of the variance is already explained by the first component.


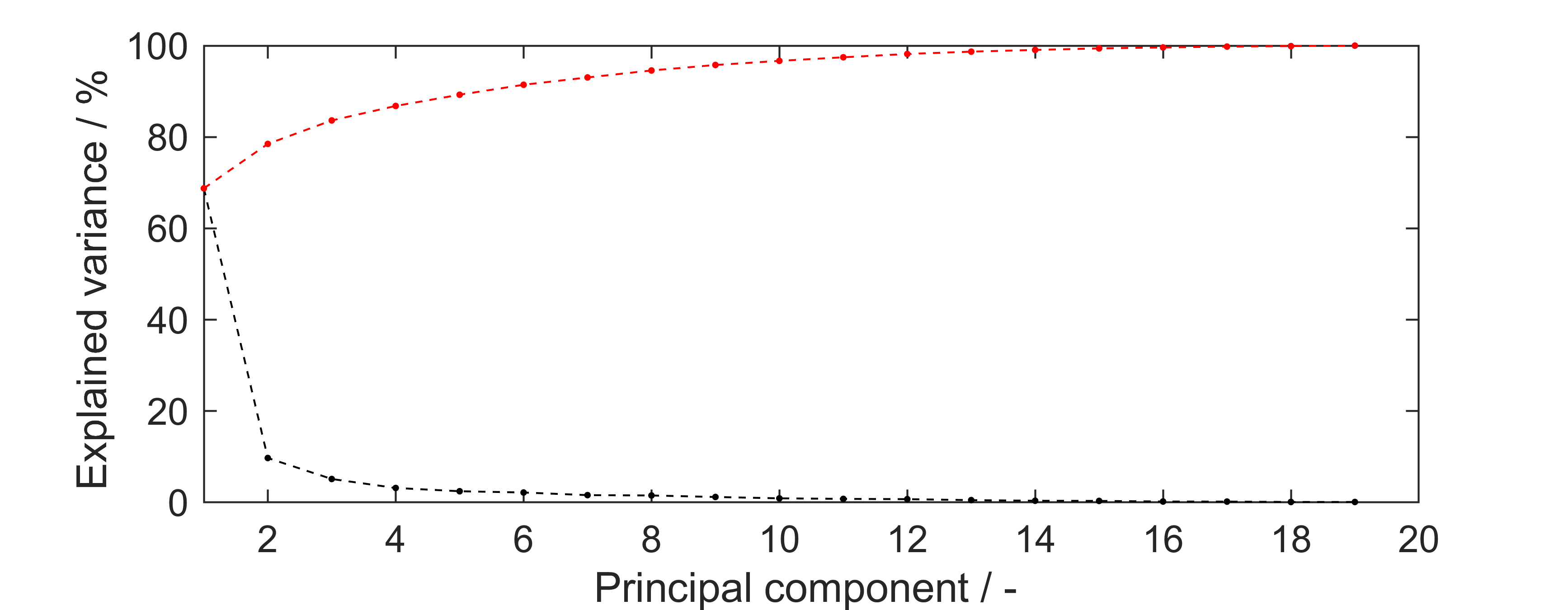


**Figure S1:** Explained variance (%) of principal components derived from 91 normalized hydrophobicity scales. The black dots represent the explained variance per principal component. The red rots represent the cumulative sum of the explained variance. Dashed lines are shown to guide the eye.

# Location and Interaction of Tryptophan within the HBcAg Dimer

Figure S2 shows a 3-D representation of HBcAg dimer backbone, with residues arginine, phenylalanine, tryptophan, and tyrosine shown and colored blue, yellow, red, and green, respectively. The four tryptophans are indicated by one-lettercode and marked with their sequence position. All tryptophans are in the vicinity of either arginine, phenylalanine, and/or tyrosine.


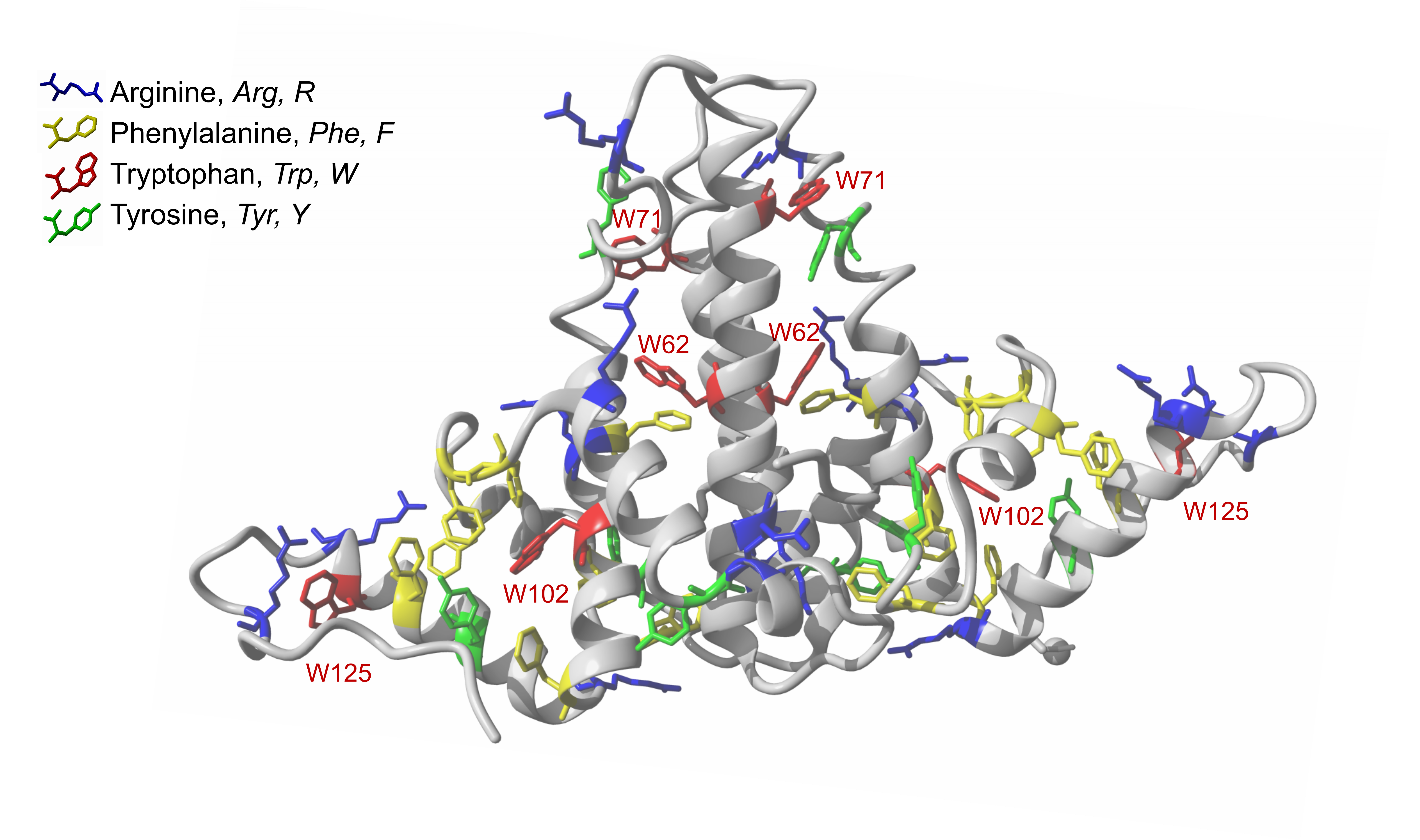


**Figure S2:** 3-D model of the truncated HBcAg crystal structure 4BMG (retrieved from [www.rcsb.org](http://www.rcsb.org), created with YASARA version 18.2.7). Four amino acids are highlighted: arginine, blue; phenylalanine, yellow; tryptophan, red; tyrosine, green. Tryptophan residues are labeled with one-letter code and their sequence positions. Each tryptophan side chain interacts either with the side chains of arginine, phenylalanine or tryptophan.

Supplementary Material References

Hebditch, Max, M. Alejandro Carballo-Amador, Spyros Charonis, Robin Curtis, and Jim Warwicker. 2017. “Protein-Sol: A Web Tool for Predicting Protein Solubility from Sequence.” *Bioinformatics* 33 (19): 3098–3100. https://doi.org/10.1093/bioinformatics/btx345.

Idicula-Thomas, Susan, Abhijit J. Kulkarni, Bhaskar D. Kulkarni, Valadi K. Jayaraman, and Petety V. Balaji. 2006. “A Support Vector Machine-Based Method for Predicting the Propensity of a Protein to Be Soluble or to Form Inclusion Body on Overexpression in Escherichia Coli.” *Bioinformatics* 22 (3): 278–84. https://doi.org/10.1093/bioinformatics/bti810.

Magnan, Christophe N., Arlo Randall, and Pierre Baldi. 2009. “SOLpro: Accurate Sequence-Based Prediction of Protein Solubility.” *Bioinformatics* 25 (17): 2200–2207. https://doi.org/10.1093/bioinformatics/btp386.

Simm, Stefan, Jens Einloft, Oliver Mirus, and Enrico Schleiff. 2016. “50 Years of Amino Acid Hydrophobicity Scales: Revisiting the Capacity for Peptide Classification.” *Biological Research* 49 (1): 31. https://doi.org/10.1186/s40659-016-0092-5.

Smialowski, Pawel, Antonio J Martin-Galiano, Aleksandra Mikolajka, Tobias Girschick, Tad A Holak, and Dmitrij Frishman. 2006. “Protein Solubility: Sequence Based Prediction and Experimental Verification.” *Bioinformatics* 23 (19): 2536–42. https://doi.org/10.1093/bioinformatics/btl623.
